# The potential role of reprogrammed glucose metabolism: an emerging actionable codependent target in thyroid cancer

**DOI:** 10.1186/s12967-023-04617-2

**Published:** 2023-10-18

**Authors:** Sai-li Duan, Min Wu, Zhe-Jia Zhang, Shi Chang

**Affiliations:** 1 Department of General Surgery, Xiangya Hospital Central South University, Changsha, 410008 Hunan People’s Republic of China; 2 Xiangya Hospital, National Clinical Research Center for Geriatric Disorders, Changsha, 410008 Hunan People’s Republic of China; 3 Clinical Research Center for Thyroid Disease in Hunan Province, Changsha, 410008 Hunan People’s Republic of China; 4 Hunan Provincial Engineering Research Center for Thyroid and Related Diseases Treatment Technology, Changsha, 410008 Hunan People’s Republic of China

**Keywords:** Thyroid cancer, Glycolysis, Target therapy, Metabolism

## Abstract

Although the incidence of thyroid cancer is increasing year by year, most patients, especially those with differentiated thyroid cancer, can usually be cured with surgery, radioactive iodine, and thyroid-stimulating hormone suppression. However, treatment options for patients with poorly differentiated thyroid cancers or radioiodine-refractory thyroid cancer have historically been limited. Altered energy metabolism is one of the hallmarks of cancer and a well-documented feature in thyroid cancer. In a hypoxic environment with extreme nutrient deficiencies resulting from uncontrolled growth, thyroid cancer cells utilize “metabolic reprogramming” to satisfy their energy demand and support malignant behaviors such as metastasis. This review summarizes past and recent advances in our understanding of the reprogramming of glucose metabolism in thyroid cancer cells, which we expect will yield new therapeutic approaches for patients with special pathological types of thyroid cancer by targeting reprogrammed glucose metabolism.

## Introduction

Thyroid cancer (TC) is an endocrine system tumor originating from follicular thyroid cells and parafollicular C cells, and its incidence rate is rising worldwide [1, 2]. Most thyroid malignancies (> 95%) are differentiated thyroid cancers (DTCs), which include papillary thyroid cancer (PTC) and follicular thyroid cancer (FTC) [3]. Lymph node metastasis is a common clinical feature of DTC, and a considerable portion of DTC is prone to early cervical lymph node metastases (approximately 20–70%), which is an important factor affecting the prognosis of DTC patients [4, 5]. Despite the widespread use of multimodality treatment (ie, surgery, chemotherapy, and radiotherapy), survival rates have not improved much over the past few decades, suggesting that new treatment options should be explored [6–8]. Therefore, we recommend seeking other effective treatments for TC. Probing the molecular mechanism of disease progress, and developing new targeted drugs remain the focus of TC research [9]. Initiation and progression of TC involves multiple genetic and epigenetic alterations, among which common mutations found in TC are point mutations in the BRAF and RAS genes as well as RET/PTC and PAX8/PPARγ chromosomal rearrangements [10, 11]. These alterations often lead to anomalies in the proliferation, differentiation and metabolism patterns of thyroid follicular cells, acting synergistically to amplify their effects on thyroid tumor development. These mutations are crucial for the abnormal activation of the MAPK and PI3K-AKT signaling pathways, which primarily regulate cell proliferation and differentiation, but also directly regulate the activity of oxidative phosphorylation, cellular glucose uptake and aerobic glycolytic processes [12, 13]. Cancer cells frequently undergo a reorganization of metabolism to promote growth, survival, proliferation and long-term maintenance [14]. For some rapidly proliferating cells and tumors, cells use glycolysis to provide cancer cells with adenosine triphosphate (ATP), nucleotides, lipids, and amino acids needed for their growth, so that even under aerobic conditions, the glucose uptake rate is significantly increased and lactate is produced, a phenomenon known as the Warburg effect [15]. This metabolic change occurs in tumors, with marked differences in glucose use between cancer cells and normal cells [16]. However, the mechanism of the Warburg effect in TC has not yet been fully elucidated. Glucose metabolic reprogramming is a primary mode of energy production in TC and has been shown to be closely associated with tumorigenesis [15]. Importantly, these metabolic adaptations appear to be responsive not only to the genotype of the tumor, but also to the biochemical microenvironment [17]. Many studies have demonstrated that glycolysis is involved in the activation of oncogenes, such as phosphatidylinositol 3-kinase (PI3K) and hypoxia-inducible factor-1 alpha (HIF-1α) in the tumor microenvironment (TME), and acts as an energetic source for cancer cells [18]. The unique hypoxic and nutrient-deficient microenvironment further leads TC cells to utilize glucose in a hypoxic manner [15].

Here, our review mainly focuses on the interactions of glycolysis in the development of TC cells. As we understand the role of glycolysis in the growth, proliferation and metastasis of TC cells, we are able to put forward suggestions for better treatment of TC indications. At the same time, we describe the factors that affect the early detection of TC to find treatment methods that can achieve better clinical results. By understanding the process of glycolysis and its relationship with tumor cells, we can further consider the targeted treatment of TC based on the glycolysis pathway in combination with clinical treatment and whether TC can be diagnosed through the relevant factors of the glycolysis process, so as to gain insight for cancer treatment.

## Insights from glycolysis in thyroid cancer

Cancer cells are well known for a series of patterns including constant proliferative signaling, growth suppressor’s avoidance, resistance to cell death, replicative immortality, high angiogenesis, reprogrammed energy metabolism, immune-mediated destruction, invasion, and metastasis, by which they can surpass normal cells’ capacity, occupy normal tissues, and even invade into surrounding or even distant area. These characteristics are largely supported by the reprogrammed energy metabolisms, which provide sufficient and instant material for cancer cells’ energy consumption and superfluous anabolism [19]. In normal conditions, cells are dependent on glycolysis rather than oxygen-consuming mitochondrial metabolism for energy supply facing short of oxygen. However, cancer cells prefer glycolysis even when oxygen is on the scene, a phenomenon first observed by Otto Warburg [20]. Such preference is shown because even though glycolysis produce less ATP per molecule of glucose, it can yield energy at a much higher rate [21]. Hence, it satisfies the high demand of cancer cells and becomes the central pathway glucose metabolism. The cancer gene mutations together with altered glycolysis, as well, promote the branches of glucose metabolism such as pentose phosphate pathway (PPP) partly because of the upregulated flux of glucose entering the PPP branch. Besides the dysregulation of glucose metabolism, other metabolism pathways also undergone great change partly ascribe to the upstream glucose metabolism alteration and partly due to the requirements of biosynthesis of biomass, such as nucleotides, amino acids and lipids [22]. Aberrant lipid metabolism, amino acids metabolism, mitochondrial biogenesis, and other bioenergetic metabolism pathways have been gradually uncovered, showing the thorough reformation of metabolism in cancer cells [23, 24].

### Differences in glycolysis between cancer and normal cells

**Fig. 1:**
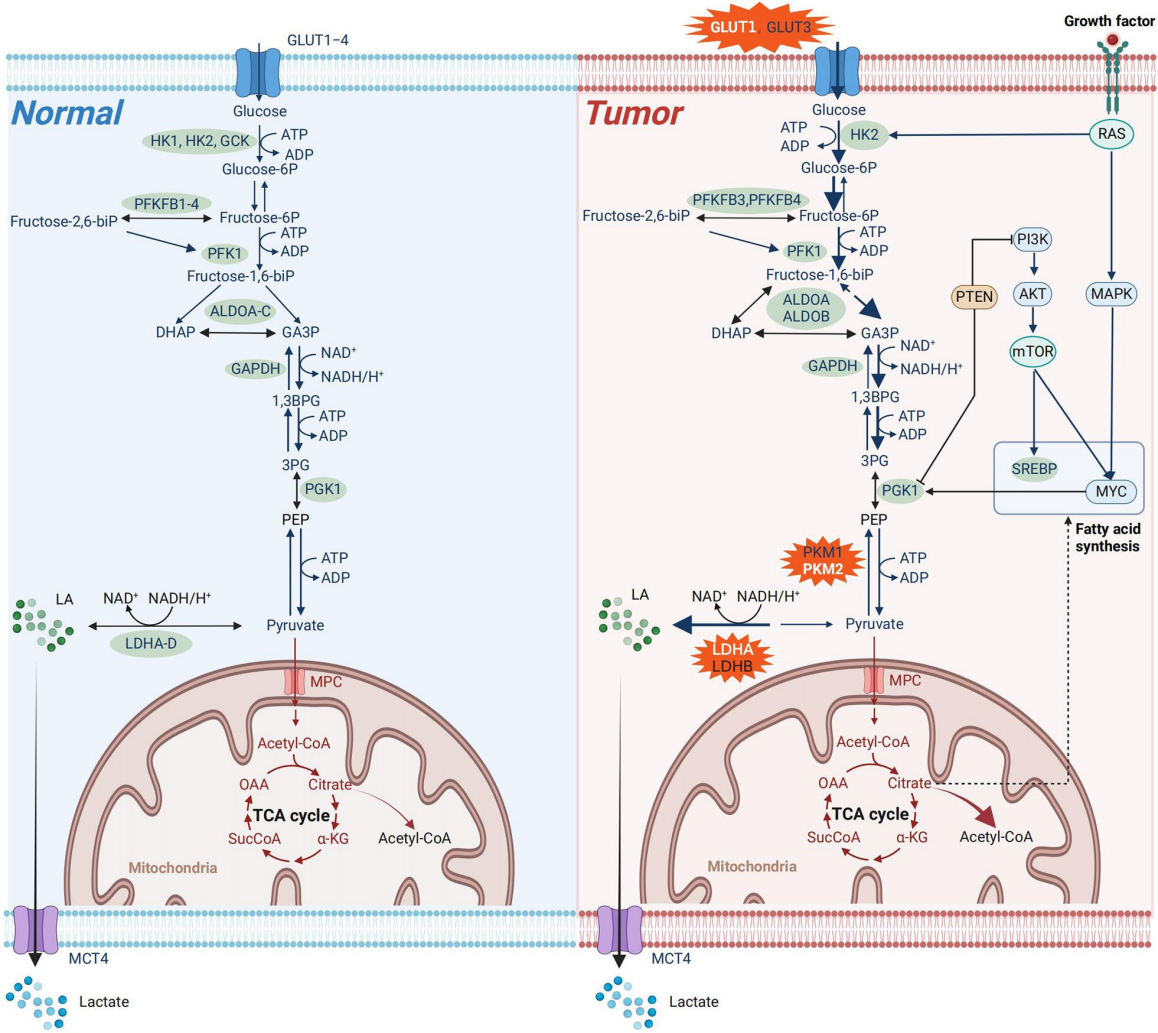
Different glucose metabolic pathways between tumor cells and normal cells. Most nonproliferating normal cells transport glucose into cells through GLUTs by acquiring oxygen molecules, which are then decompose through glycolysis and the TCA cycle. In the last step of glycolysis, the existence of pyruvate kinase M1 isoforms ensures that the product pyruvate is transported to mitochondria, where it is then oxidized in the process of PDH to produce acetyl coenzyme A and enter the TCA cycle. In tumor cells, GLUT1 and 3 transport a large amount of glucose into the cytoplasm for glycolysis even in tumor cells with adequate oxygen supply. It relies on the pyruvate kinase M2 isoform to convert pyruvate into the substrate of LDHA, producing a large amount of lactic acid and secreting the extracellular matrix. Since only a small amount of glucose is transported to the mitochondria for decomposition, each glucose molecule is decomposed to fewer ATP molecules. GLUT, glucose transporter; HK, hexokinase; GCK, glucokinase; ATP, adenosine triphosphate; ADP, adenosine diphosphate; Glucose-6P, Glucose 6-phosphate; Fructose-6P, fructose-6-phosphate; PFKFB1-4, 6-phosphofructo-2-kinase/fructose 2,6-bisphosphatase; Fructose-1,6-biP, fructose-1,6-bisphosphate; Fructose-2,6-biP, fructose-2,6-bisphosphate; PFK1, phosphofructokinase 1; ALDO, aldolase; DHAP, dihydroxyacetone-phosphate; GA3P, glyceraldehyde 3-phosphate; GAPDH, glyceraldehyde- 3-phosphate dehydrogenase; GAPDH, Glyceraldehyde 3-phosphate dehydrogenase; 1,3BPG, 1,3-Bisphosphoglyceric acid; 3PG, 3-Phosphoglyceric acid; LDH, Lactate dehydrogenase; MPC, 2-methacryloyloxyethyl phosphorylcholine; OAA, oxaloacetate; Suc-CoA, Succinyl-CoA; α-KG, α-ketoglutarate; Acetyl-CoA, acetyl coenzyme A; MCT4, MCT, monocarboxylate transporter 4; TCA, tricarboxylic acid

There are three main differences in the glycolysis process in TC cells compared with normal cells (see Fig. 1), such as glucose transports, pyruvate kinases, and lactic acid metabolism. In terms of glucose transport, TC has a high demand for glucose, so it overexpresses glucose transporters to transport a large amount of glucose through the membrane. In this process, controlling the expression of pyruvate kinase (PK) will block the final step of glycolysis, leading to the accumulation of many early intermediate metabolites in tumor cells. Otherwise, enhanced glycolysis and reduced oxygen consumption in cancer cells would lead to the expansion of lactate.

#### Glucose transport

Excessive proliferation is one of the main differences between cancer and normal cells. Therefore, a common characteristic of metabolic changes in tumor cells is increased glucose uptake [25], which can also be observed when mitochondrial functions are complete [26–28]. To meet the large nutritional demands in the course of cell proliferation, tumor cells adopt a very uneconomic way of glucose metabolism to ensure that a large amount of glucose enters the cells for decomposition [29]. TC cells usually exhibit a state of hypoxia, which prevents the cells from performing sufficient glycolysis and providing sufficient ATP [30]. However, tumor cells are well adapted to this hypoxic environment [31] due to glycolysis. This behavior has been observed in all sorts of tumors [32]. A defining feature of TC cells is their ability to absorb large amounts of glucose compared to normal thyroid tissues. The upregulation of glucose transporters (GLUTs) has been reported to be an indicator of aggressiveness and loss of tumor differentiation in TC [33]. In most cases, TC cells tend to exhibit GLUTs overexpression, particularly the hypoxic-reactive GLUT1 and GLUT3 proteins [34]. The primary cellular function of the GLUT is to facilitate the entry of glucose molecules into cells [35]. Among GLUTs, GLUT1 is the most frequent isoform in many cancers, such as lung cancer, colorectal cancer, prostate cancer, and hepatocellular carcinoma [36–39]. In several cancers, overexpression of GLUT1 is related to invasion and poor survival [40, 41], while increased GLUT1 expression improves glycolysis [42]. Previous studies have demonstrated that the translocation of GLUT1 to the cancer cell membrane is a factor limiting the rate of cellular energy generation [42]. The high expression of GLUT1 in TC is positively correlated with the proliferation index, which is equivalent to malignant characteristics [43]. In particular, overexpression of GLUT1 on cell membranes correlates perfectly with the rate of cell de-differentiation and greater biological aggressiveness of TC [44]. Greater GLUT1 expression can often be detected by immunostaining in TC, but not in benign nodules or normal thyroids [45]. This suggests that GLUT1, as a rate-limiting step in glucose metabolism in cancer cells and a modulator of glucose uptake pathways, is a promising target for the development of anti-cancer strategies.

#### Pyruvate kinase and pyruvate carboxylase

TC cells consume more glucose compared to normal cells [46], limiting the final step of the glycolysis pathway through their negative feedback mechanism and thus, leading to the accumulation of many early intermediate metabolites in tumor cells [47]. Even in a tumor microenvironment with normal oxygen levels, TC increases glucose absorption, metabolizes glucose to acrylic acid, and then converts the product to lactic acid (LA) rather than allowing it’s to enter the TCA cycle [48]. Pyruvate kinase (PK) facilitates the last step of glycolysis, the exchange of phosphoenolpyruvate (PEP) with pyruvate and is involved in the TCA cycle. The intersection between anabolic and catabolic pathways is primarily conducted by PK [49], mainly PKM1 and PKM2. PKM1 regulates the transport of pyruvate from the cytoplasm to mitochondria, while PKM2 regulates the decomposition of pyruvate to LA in the cytoplasm of tumor cells [50]. Compared with PKM1, the PKM2 isoform has a low catalytic enzyme efficiency, leading to the accumulation of glycolytic intermediates, and is involved in other biochemical synthesis pathways [51–53]. During PKM1 activation, anabolic synthesis (or branching pathways in glycolysis) is promoted [54, 55], while phosphoenolpyruvate (PEP) is converted to pyruvate due to PKM2 activation, to produce ATP molecules [49, 56]. The activity of the PKM2 tetramer promotes the complete oxidative decomposition of glucose into ATP through oxidative phosphorylation, while the activity of its dimer promotes the glycolysis [57]. Some studies have suggested that PKM2 is related to the poor prognosis and has been identified as a prognostic marker in many cancers [58, 59]. One study has stated that PKM2 is involved in the progression of TC [60]. PKM2 is significantly overexpressed in PTC, especially in cases harboring BRAF mutations, and its overexpression is closely related to advanced tumor stage and lymph node metastasis [60]; meanwhile, PKM2 knockdown significantly inhibits PTC cell growth, lactic acid and ATP production, and glucose consumption [61]. Additionally, the activation or up-regulation of PKM2 could activate multiple cancer-related pathways such as ERK signaling and STAT3 signaling [62, 63]. Therefore, inhibition of PKM2 may be potential to inhibit glycolysis and thus the proliferation of tumor cells. Moreover, pyruvate carboxylase (PC), a key enzyme at the intersection of glycolysis and the TCA cycle in TC cells, plays an important role in replenishment [64]. It is reported that PC is strongly involved in the tumor aggressiveness of TC via its stimulation of fatty acid synthesis [65]. Hence, PC restraint can significantly reduce TC cell proliferation [65], suggesting that it may be possible to detect the expression of PC in living tissues to reflect the invasive behavior of tumors and provide valuable information for clinical diagnosis and treatment of TC.

#### Lactic acid metabolism

As a key energetic source, a glucose precursor, and a signal molecule, LA plays a vital role in shuttling between cells in vivo [66]. Previous studies have shown that glycolysis plays a role in cell signal transduction [67, 68], promoting proliferation, invasion, and drug resistance in cancer cells [69]. Aberrant glycolysis in TC involves increased LA production and accumulation, which further promotes pH conditions conducive to growth and invasion [70]. Lactate dehydrogenase (LDH), which includes two isoforms (LDHA and LDHB), plays a decisive role in LA production. LDHA is responsible for converting PA into LA and NAD, whereas LDHB converts LA into PA and promotes oxidative metabolism [66]. The increase of LDH activity leads to tumor immune evasion via inhibiting the function of immune cells [71]. For instance, LDHA-associated LA accumulation in melanoma has been shown to inhibit tumor monitoring by T and NK cells [71]. LDHA can increase acetylation and transcription of interferon-γ (IFNG) to promote T cell effector functions, thereby highlighting the key role of LDH in inflammation [72]. Changes in LDHB expression are often associated with early metabolic adaptation [73]. LDHB-mediated LA use supports autophagy to maintain metabolic health and cancer cells growth [74], indicating that the production and use of LA may be involved in the metabolic adaptation of cancer cells to support the development of metastasis. For example, overexpression of LDHB significantly inhibits the inhibitory effects of HYOU1 silencing on aerobic glycolysis, proliferation, migration, and invasion of PTC cells [75]. In glycolytic tumors, LA levels in cancer cells are increased more than 40-fold and are highly correlated with cancer invasion and low survival rate [76, 77]. Inhibition of the mitochondrial biogenesis pathway will decrease tumor survival and reduce tumor progression [78]. LA inhibits the differentiation of monocyte into dendritic cells [79], suggesting that high LA levels in the TME may hinder the formation and accumulation of dendritic cell. Meanwhile, high LA levels in the TME also inhibit LA efflux from T cells, resulting in decreased cytokine production and cytotoxic activity [80]. Inhibition of LA shuttle has been reported to significantly reduce the proliferation and glycolytic capacity of ATC cells in a low-glucose environment [81]. TC cells rely on glucose to activate the PI3K pathway, which influences many cellular processes, such as metabolism, cancer progression, and metastasis [82]. PI3K signaling can regulate GLUT1 expression through Akt, enhance glucose intake and facilitate phosphofructokinase (PFK) activity [83] to further promote the increase in LA. Thyroid oncogene mutations, such as c-Myc, can also increase GLUT1 expression in cancer cells, affecting glucose metabolism, and driving cell malignant transformation [84]. Therefore, glycolysis can help cancer cells survive, grow, and metastasize and further help cells resist apoptosis and avoid immune system destruction [85]. The expression of LA and LDH can support the metabolic adaptation and tumorigenesis of cancer cells [81]. Therefore, targeted suppression of glycolytic and lactate processing pathways may represent an effective treatment strategy for TC.

### The role of glycolysis in thyroid cancer

Abnormal glycolysis in TC can acidify the tumor microenvironment, further leading to the abnormal growth of cancer cells. Acidification leads to changes in biological factors in the environment, which can promote or inhibit further development of TC. The fatal element of TC is metastasis, which is also affected by alterations in the tumor microenvironment also affects tumor metastasis [86].

#### Thyroid cell carcinogenesis and tumor formation

A necessary condition for cell growth is energy supply, and glycolysis is a crucial method to provide power for cell growth. Compared with normal cells, tumor cells have inefficient energy production, which implies that their growth and reproduction require more glucose to provide power [87]. Tumor cells can exhibit a specific metabolic pattern, which can quickly transport and consume glucose to produce ATP and boost drug excretion [88]. Meanwhile, increased levels of reactive oxygen species (ROS) are also an essential feature of TC cells, and high ROS production may lead to cell damage and cell death [89]. ROS have been demonstrated to play a significant role in cell proliferation, metabolism, angiogenesis, cell growth, and survival in several advanced malignant tumors [90, 91]. In TC, cancer cells preferentially undergo glycolysis even under aerobic conditions [92]. Many genes are upregulated or downregulated to change glycolysis, thereby promoting or inhibiting tumor growth (shown in Table 1).

**Table 1:** Regulators of glycolysis associated with thyroid cancer cells growth

Regulators		Effects in glycolysis	Effects in TC growth	Downstream molecules	Participation pathway	Mechanism
Negative regulators	PTEN	Negative	Negative	HIF-1, VEGF, PCNA	PI3K/PTEN/AKT, PI3K-AKT-mTOR	Inhibit GLUT1 expression and glucose uptake in TC, downregulate PI3K-AKT-mTOR pathway and affect glucose metabolism
	P53	Negative	Negative	AMPK, GLUT1,3,4, PGM, TSC2, RRAD	PI3K-AKT-mTOR, caspase pathway	Shorten glucose uptake and promote mitochondrial oxidation, so as to resist Warburg effect, which also leads to cell cycle arrest and apoptotic cell death
	Iodide	Negative	Negative	GLUT1	Oxidation pathway, rate-limiting glucose-facilitated transport system	Inhibit TSH induced stimulation of glucose transport, reduce the number of available carrier sites and inhibit cell growth
	BRAF^V600E^	Negative	Negative	GLUT1	RAF/MEK/ERK	Initiate the glycolytic table associated with GLUT1 overexpression and inhibit mitochondrial respiration in thyroid cells
Positive regulators	HIF-1	Positive	Positive	GLUT1, PDK, PKM2, HKII	PI3K/AKT	Enhance glycolysis, increase GLUTs expression, and promote tumor growth
	PI3K/AKT	Positive	Positive	GLUT1, HKII, PDK1	PI3K/AKT	Promote cell carcinogenesis and increase glycolytic flux
	TSH	Positive	Positive	mTOR	PI3K.AKT, RAS/MAPK	Promote thymocyte proliferation and thyroid proliferation
	c-Myc	Positive	Positive	GLUT1, LDHA, PK, PKM2, MCTs	APC, miR-222-3p/HIPK2/ERK	Promote anaerobic glycolysis, tumor growth and cell proliferation
	AMPK	Positive	Negative	HIF-1α, mTOR	AMPK/AKT, AMPT/mTOR	Regulate glycolysis and control cell growth, apoptosis and survival
	LDHA	Positive	Positive	STAT3	JAK/STAT	Promote the conversion of pyruvate to lactic acid, so as to promote the glycolysis process and tumor growth
	PD-1	Positive	Positive	SHP2, RAS	SHP2/RAS/MAPK, RAS-MAPK-ERS	Promote the proliferation and vitality of thyroid cancer cells

BRAF^V600E^ mutations are common in TCs [93, 94]. The BRAF^V600E^ mutation can alter the HIF1-Myc-PGC1 axis, leading to inhibition of mitochondrial respiration and enhancement of aerobic glycolysis [95]. Meanwhile, glycolytic enzymes (such as LDHA and PKM2) are regulated by HIF1 and Myc to promote glycolysis, and BRAF^V600E^ can regulate phosphate MEK1/2, thereby reducing mitochondrial metabolism [95]. The role of BRAF^V600E^ signaling in the regulation of tumor metabolism suggests that BRAF can generate biodynamic adaptation by inhibiting oxidative phosphorylation [96]. Other studies have shown that glucose restriction in the cellular environment can restrain the proliferation of ATC cells [97, 98], while programmed cell death protein 1 (PD-1) can promote the proliferation and viability of TC cells [99]. HIF1 inhibits mitochondrial respiration and Myc activity in TC by inhibiting the expression of peroxisome proliferator-activated receptor γ coactivator-1 (PGC-1), indicating that the metabolic reprogramming may be a key step in thyroid carcinogenesis [100].

Mutations in the RAS-MAPK-ERK and PI3K-Akt-mTOR pathways usually exist in highly differentiated tumor components, and most ATCs are developed from these tumor components [101, 102]. In ATC, genetic alterations in the p53 gene are the most common (55%) changes [103]. Approximately 40% of PTC and 22% of FTC have p53 gene changes [104]. Studies have found that pAKT is highly expressed with pERK and low in PTEN in ATC patients, which indicates that the two pathways of RAS-MAPK-ERK and PI3K-AKT-mTOR play a synergistic role in the development of ATC [99]. In addition, the mutation of p53 was negatively correlated with the expression of pAKT, and there was a significant positive correlation between PTEN and pERK [99]. Activation of the PI3K-AKT-mTOR signaling pathway inhibits ERK1/2 activation, which suggests that the RAS-MAPK-ERK or PI3K-AKT-mTOR pathway controls the carcinogenic effects of ATC [105]. mTOR mediator signals are combined with PKB/Akt, HIF1, and AMPK signaling pathways to manage cell proliferation and survival under conditions of nutrient and energy deprivation [106]. mTOR is a central activator of the Warburg effect [2]. mTOR upregulates PKM1 expression through mediated transcriptional activation of HIF1α and c-Myc heteroribonucleoprotein-dependent regulation of PKM2 gene splicing [107]. The destruction of PKM1 inhibits oncogenic mTOR mediated tumorigenesis [108]. Unlike normal cells, mTOR hyperactive cells are more sensitive to the inhibition of mTOR or glycolysis. The dual inhibition of mTOR and glycolysis synergistically passivates the proliferation and tumor development of mTOR hyperactive cells [107]. PD-1 can activate Ras-MAPK signaling cascade in TC cells and enhance the expression of Ras in TC cells [109]. In addition, RET/PTC or BRAF mutations can also lead to active PI3K [110]. In TC cells, downregulation and activation of the Ras-MAPK and PI3K-Akt pathways mainly inhibit cell migration and proliferation [111]. However, inactivation of Ras-MAPK signaling has a positive effect on the mobility of ATC cells [112]. Compared to the inhibition of a single pathway, the dual Ras-MAPK and PI3K-Akt-mTOR pathways can inhibit cell growth and even lead to growth retardation in TC cells in a congenerous manner [113]. In addition to the influence of these factors and pathways, glycolysis leads to acidification of the tumor microenvironment, which also promotes TC progression.

#### Glycolysis and thyroid cancer microenvironmental acidosis

**Fig. 2:**
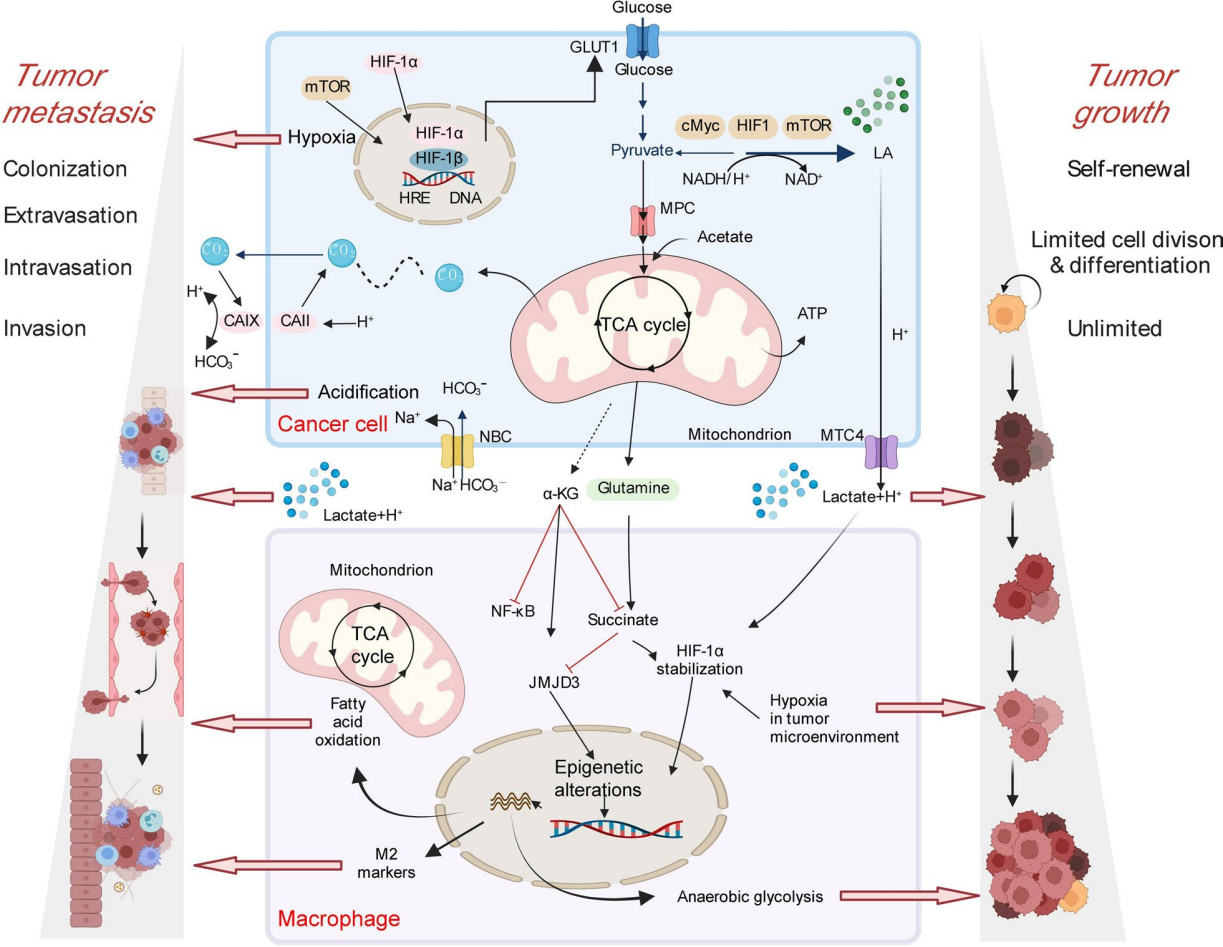
Anaerobic glycolysis promotes the growth and metastasis of thyroid cancer cells. Anaerobic glycolysis of tumor cells produces a large accumulation of lactic acid, which acidifies the tumor microenvironment. Tumor growth mainly consists of three steps: self-renewal, limited cell division or differentiation, and an unlimited state. Tumor metastasis includes colonization, extravasation, intravasation, and invasion. A hypoxic environment, excessive lactic acid and anaerobic glycolysis can promote the rapid growth and metastasis of tumor cells. cMyc, HIF1α, and mTOR can promote the formation of lactic acid and the expression of GLUT1 to help tumor growth. The simultaneous hypoxia can promote tumor metastasis. The increased influx of sodium ions in tumor cells increases the level of HCO3-, further promoting tumor microenvironmental acidification and metastasis. Fatty acid oxidation in macrophages in the tumor microenvironment and M2 markers can also promote tumor cell migration. The glutamine produced by tumor cells can promote the production of succinate, thereby making HIF1α stabilization, which further promotes the hypoxia of the tumor microenvironment, so that tumor cells undergo anaerobic glycolysis, thereby assisting tumor cell growth and metastasis

Glucose is converted to LA in tumor cells and flows extracellularly to form lactate and produce lactate accumulation (Fig. 2) [114]. Through continuous aerobic glycolysis, glucose alters some of the microenvironment, resulting in side effects [115]. LDHA of the glycolysis process promotes the conversion of pyruvate to LA, which is associated with the development of various cancers, including TC [116–118]. Proton-linked monocarboxylate transporters (MCTs) transport LA across the plasma membrane, which requires binding of CAIX to the CD147, a widely expressed membrane glycoprotein [119]. Studies have shown that MCT1 is required for CD147 protein expression, causing the MCT/CD147 subunits to assemble and target the plasma membrane [120]. In rat thyroid tissues, MCT4 can output LA through the plasma membrane with the assistance of CD147 [120]. Research has found that Acriflavine (ACF) can disrupt the binding of MCT4 to its essential cofactor basigin [121]. ACF can effectively inhibit the growth of ATC cells in vitro by inhibiting LA output and subsequently inhibiting upstream glycolysis [81]. High levels of acid production result in a sharp local drop in the extracellular pH value. In addition to lactate, carbon dioxide (CO_2_) manufactured by catalytic pathways, such as the pentose phosphate pathway (PPP), is also conducive to acidifying the TME [122, 123]. In this case, microenvironmental acidification can promote tumor invasion by destroying adjacent normal cells, inducing extracellular matrix (ECM) degradation, and promoting angiogenesis [114]. Long-term exposure of normal cells to an acidic microenvironment leads to cell necrosis or apoptosis depending on p53 and caspase-3 mechanisms [124]. Nevertheless, tumor cells adjust their survival conditions to adapt to the acidic microenvironment [125]. The acidic microenvironment can inhibit the growth of normal cells, but acidosis is an indispensable criterion for cancer cell migration and invasion [124]. High levels of LA in TME will reduce the activity of immune cells, thereby promoting tumors and metastasis [126, 127]. Meanwhile, the accumulation of lactate and acidification of TME will accelerate the remodeling of basement membranes (BM) or boost the progression of epithelial-mesenchymal transition (EMT), contributing to tumor invasion [128]. Acidosis can affect tumor progression, aggression and metastasis, a phenotypic feature of TME markers [129]. The glucose uptake of tumor cells increases due to hypoxia, and glucose restriction in the TME also facilitates the activation of the M2-like phenotype in tumor-infiltrating macrophages, promoting the anti-inflammatory response and tumor growth [130]. In conclusion, aerobic glycolysis in tumor cells produces many lactic acid accumulations, which acidifies the TME, destroys adjacent normal tissues, degrades the extracellular matrix, and promotes angiogenesis, thus promoting tumor invasion and metastasis.

#### Glucometabolic reprogramming in metastatic thyroid cancer

Tumor metastasis to distant organs is caused by tumor cells with primary heterogeneous tumor diffusion, and the sequential growth and survival of tumor metastasis depend on different metabolic changes [131]. Malignant tumors proliferate indefinitely and have a tendency for distant metastasis. They require large amounts of energy and biosynthetic precursors to promote cell division, invasion, and migration [132, 133]. Secreted lactic acid can affect cell types in TME by activating multiple processes such as tumor cell survival and proliferation [134]. LA accumulation can induce various events in the TME, including the upregulation of hyaluronic acid, which is conducive to tumor migration [135, 136]. Lactate excretion by tumor cells allows acidic degradation of the matrix around healthy tissues, leading to invasive growth [137]. Tumor metastasis is a multistep cascade process, and more than 90% of cancer deaths are not caused by tumors alone, but by tumor metastasis [138]. At the beginning of metastasis, invasion is required. That is, diffuse malignant cells must converge the normal ambient tissues into a collective tissue structure or separate into small cell clusters [139, 140]. As tumor cells reduce cell-to-cell adhesion to relax tight structures, they promote further cell invasion, which is a feature of the EMT process [141, 142]. During the EMT process, the viscosity of tumor cells decreases and the activity of tumor cells increases. After converging normal ambient tissues and forming a new vascular network by secreting vascular factors, tumor cells will connect the small blood vessels, such as veins or capillaries, and lymph nodes and enter the circulation [143]. Interaction with neutrophils in circulation can promote further metastasis and diffusion. Neutrophils may also promote tumor cell extravasation by secreting matrix metalloproteinases (MMPs) [144]. Eventually, tumor cells can leave the blood circulation and invade secondary tissues.

**Fig. 3:**
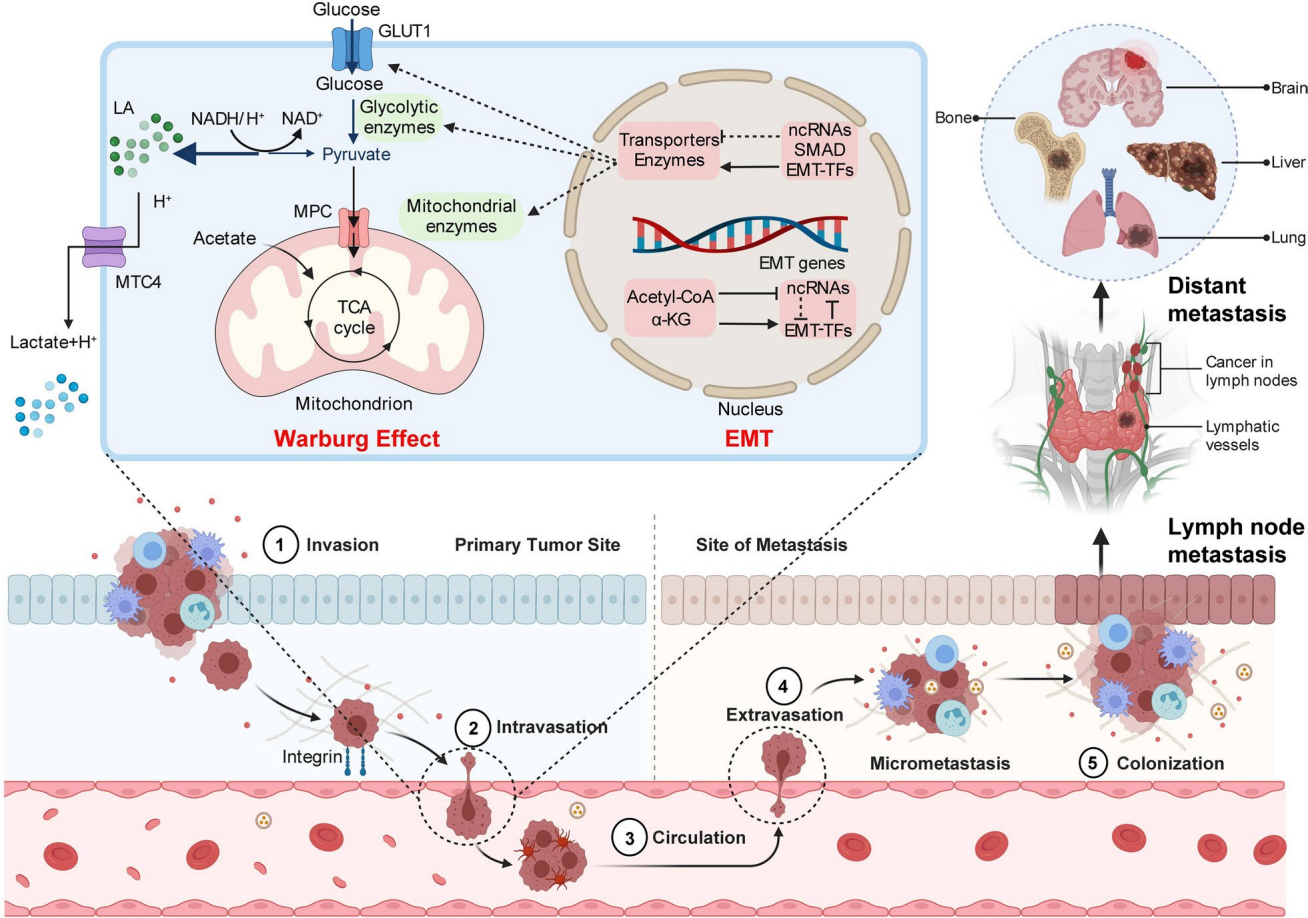
Relationship between metastasis of thyroid cancer cells and glycolysis and EMT. Metastasis of thyroid cancer cell is a multistage process including invasion, intravasation, circulation, extravasation, and colonization. Intravasation and extravasation are closely related to glycolysis and the EMT process. One of the reasons for the extremely high risk of thyroid cancer is that it can metastasize remotely through lymph nodes, often to the lungs, bones, liver and brain

Metastasis occurs in 10% of TC patients (Fig. 3), and approximately half of distant metastases occur in the lung, which may be associated with anti-nest loss apoptosis and pro-invasion signals mediated by LDHA phosphorylation [145, 146]. LDHA phosphorylation provides invasive signals in metastatic cancer cells by regulating redox status, and LDHA can also enhance tumor progression by possessing molecules related to EMT [147]. When LDHA is inhibited, more pyruvate will enter the tricarboxylic acid (TCA) cycle, resulting in an increased oxygen demand [147]. However, cancer cells are overdependent on aerobic glycolysis, which produces ATP rapidly and can use more precursors to meet the metabolic requirements of rapid proliferation. Therefore, when LDHA is inhibited, it can affect the proliferation, invasion, and metastasis of TC cells and prevent TC cells from escaping immunity [100]. In conclusion, LDHA can cause EMT-like changes that promote migration and invasion of TC cells, and can therefore be considered as a target factor for the treatment of TC. Increased GLUT-1 expression is also related to the increased invasive behavior and metastasis characteristics [148, 149]. HIF1, a downstream target of GLUT1, is also involved in tumor metastasis and migration [150]. Under hypoxiaconditions, increased glucose uptake by cancer cells can upregulate the stability of HIF-1α, leading to a weakened antitumor immune response [18]. In addition, programmed death-ligand 1 (PD-L1) is a downstream target of HIF-1 that can bind to PD-1 on T cells. The PD-1/PD-L1 interaction can activate the dephosphorylation of PI3K and block the Akt/mTOR pathway [151, 152].

## Potential clinical value of glycolysis in thyroid cancers

### Tools for detecting thyroid glycolysis

At present, many methods are available to help diagnose TC, and each of these methods has its own advantages and disadvantages, such as ultrasonography, fine-needle aspiration biopsy (FNAB), computed tomography (CT), and magnetic resonance imaging (MRI) (See Table 2). Among them, the diagnostic method of fluoro-2-deoxy-D-glucose positron emission tomography (FDG-PET) imaging is closely related to the glycolysis pathway. Taking advantage of exploiting the high activity of GLUT1 in tumor cells, FDG-PET imaging accumulates a significant amount of glucose in tumor cells and conducts in vivo detection in humans [153]. The diagnosis of malignant tumor metastasis relies heavily on this technology. In the thyroid gland, follicular epithelial cells exhibit fanatical iodine absorption mediated by the Na^+^/I^−^ symporter (NIS) [154]. Glucose uptake is increased by GLUT1 overexpression during differentiation in TCs. The opposite relationship between iodine absorption and glucose utilization is known as the iodine/FDG ‘turnover phenomenon’, reflecting the cell differentiation state and heterogeneous pattern of NIS expression [155, 156]. FDG-PET imaging is not recommended for the evaluation of patients with newly identified thyroid nodules or thyroid diseases [156]. Nevertheless, skeletal muscle metastasis of thyroid microcarcinoma can be evaluated by 18F-FDG PET/CT [157]. 18F-fluoro-2-deoxy-D-glucose (18F-FDG) is the most commonly used radiotracer in oncology imaging for staging, re-documentation, and assessment of treatment response in several tumors [158]. In DTC patients, lesions with high 18F-FDG and low radioactive iodine uptake are more clinically invasive [159]. Studies have found that malignant cells have the lowest degree of differentiation and the highest ability to absorb 18F-FDG [160]. Quantitative 18F-FDG-PET/CT evaluation can exclude the malignancy of uncertain thyroid nodules [161]. 18F-FDG-PET/CT can also be used to evaluate response to treatment, detect lesions in metastatic patients, and predict the prognosis of high-risk patients [162]. Although the evaluation of node status has reasonable specificity (94%), 18F-FDG PET/CT imaging shows a low sensitivity (30%) [162, 163], and the current American Thyroid Association (ATA) criteria do not recommend 18F-FDG PET/CT as a routine preoperative test [164]. However, 18F-FDG PET/CT is strongly recommended for follow-up of high-risk patients with elevated serum thyroglobulin (Tg) and negative 131I imaging [164, 165]. FDG PET has been shown to be helpful in detecting persistent or recurrent DTC in patients with low Tg; however, when FDG PET-CT is negative, this does not exclude DTC and requires further investigation [166].

**Table 2:** Detection strategies in thyroid cancers

Method	Principle	Frequency	Advantages	Disadvantages
Ultrasonography	Use ultrasound to present the internal image of opaque objects	Always	Detection of residual thyroid cancer in cervical lymph nodes or soft tissue	Unclear imaging, difficult qualitative, inaccurate quantitative
FNAB	A sterile puncture needle was used to puncture the suspicious part of the nodule, and some nodule components were extracted for cytological and pathological examination	Always	Differential diagnosis between benign and malignant thyroid nodules and diffuse goiter	Too few materials to know whether the blood vessels and capsule are invaded at the same time
CT	The optical signal is changed into an electrical signal, then into a digital signal, and finally into a computer picture	Always	Preoperative staging, monitoring, re staging, location of metastatic disease and continuous monitoring of progression and treatment response of thyroid cancer	Difficult to find small lesions with little or no density change
MRI	Based on the low diffusion coefficient of water molecules in high cell tissues	Often	Helpful to detect lymph node involvement and lymph node metastasis before operation	Expensive equipment, long time to image and limited patients
Radioiodine imaging	TSH stimulates iodine uptake in residual normal and malignant thyroid tissues	Often	Identify, locate and monitor the progress or treatment response of iodine preference metastasis in differentiated thyroid cancer	Needed TSH to stimulate iodine uptake in residual normal and malignant thyroid tissues
^123^Ι/^131^Ι/^99m^Tc Thyroid Scintigraphy	Effective concentration of iodine based on thyroid follicular cells	Often	The only evidence of autonomic functional thyroid nodules	Uncertain to hyper-functional nodules
FDG-PET	Based on the mutual annihilation of positrons and electrons, two high-energy 511 keV photons are released in the opposite direction	Often	Evaluation of thyroid cancer recurrence and for systemic and focal dosimetry	Limitation for patients with newly discovered thyroid nodules or thyroid diseases were evaluated

Diffusion-weighted imaging (DWI) provides quantitative and qualitative information based on the assessment of micro movement of water at the cell level, and can be used to distinguish benign and malignant diseases [167]. ATA states that cervical ultrasound is the best method to assess the status of lymph nodes prior to surgery [167]. MRI is a sensitive imaging modality that localizes sites of potential recurrence of DTC in the neck, mediastinum, bones, and liver, although the accuracy of detecting lung lesions is low [168]. MRI significantly reduced the total radiation dose of patients compared to PET/CT [169]. Meanwhile, PET/MRI is a promising tool with great potential to provide complementary data obtained under the same time and conditions. Diagnosis of thyroid nodules by conventional ultrasound relies on image quality, neck coverage, and ultrasound interpretation [170]. The current gold standard for confirming the diagnosis of TC is FNAB, but it remains highly likely to fail to describe micronodules of the thyroid gland [171]. Therefore, it is important to combine the available tools, such as ultrasound, CT and MRI, to establish a correct diagnosis of TC and evaluate the curative effect after treatment.

### Therapeutic strategies targeting glycolysis dependence in thyroid cancers

**Fig. 4:**
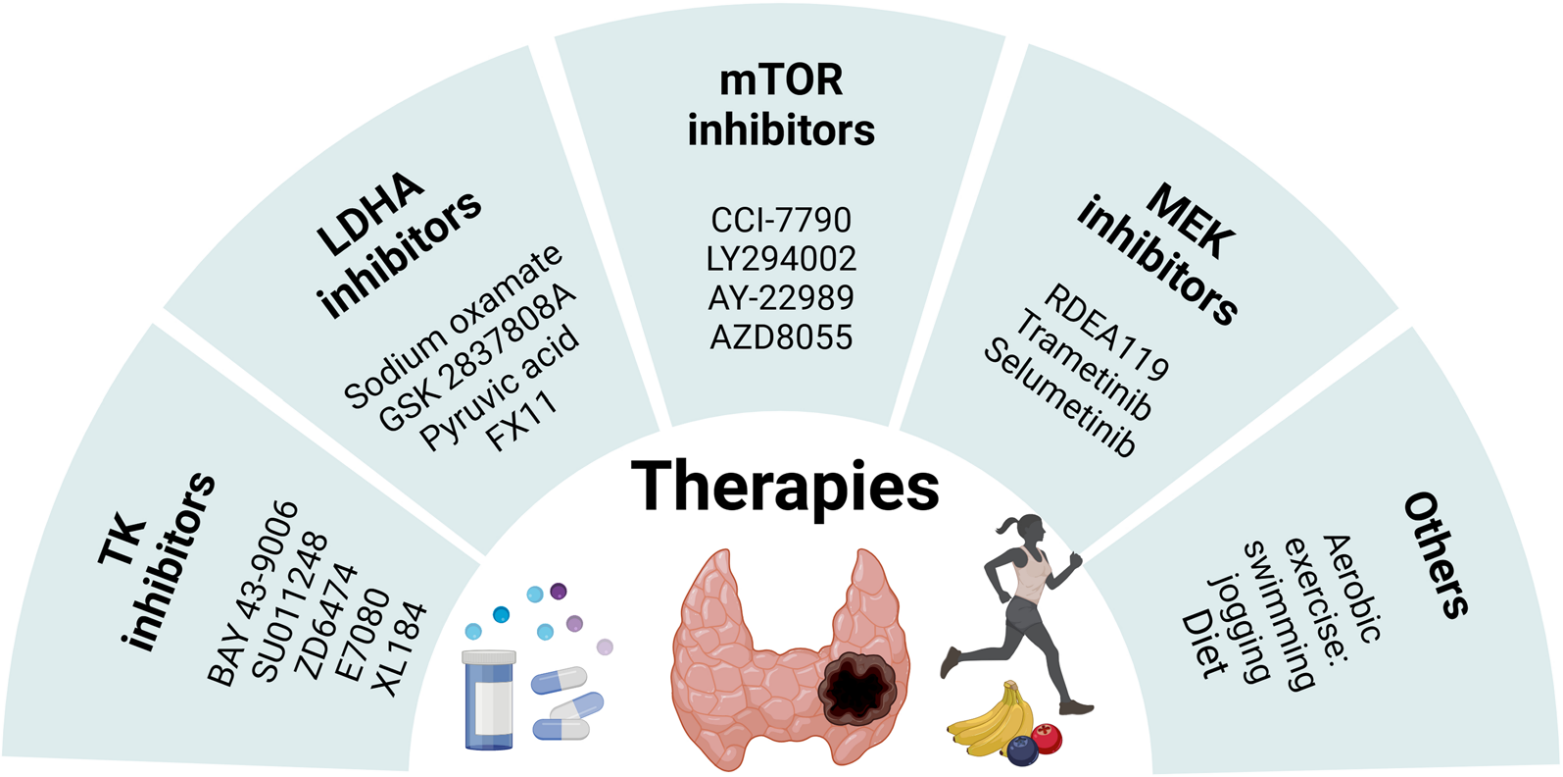
Therapies targeting thyroid cancer based on crucial factor inhibitors of glycolysis. Four inhibitors associated with glycolysis can serve as prospective treatment methods for thyroid cancer: TK inhibitors, LDHA inhibitors, mTOR inhibitors, and MEK inhibitors. Additionally, aerobic exercise might be a new strategy to reduce the incidence of thyroid cancer

Glycolysis brings many advantages to fast-growing tumor cells [172], and targeting metabolic pathways may be a promising method for tumor therapy [173]. Many targeted treatment methods are available for TCs, but the existing techniques are not systematically integrated. Among them, treatment strategies targeting glycolysis have received considerable attention. Below, we summarize some specific targeted treatment methods for TCs based on crucial factors of the glycolysis process (Fig. 4).

#### Tyrosine kinase inhibitors (TKIs)

Tyrosine kinase receptors are involved in cancer proliferation, angiogenesis and lymphangiogenesis [8, 174]. Angiogenesis plays a significant role in the occurrence and development of tumors, while lymphangiogenesis is critical for metastasis formation [175]. The expression of VEGF in TC cells can facilitate tumor angiogenesis [176]. Vascular endothelial growth factor receptor 2 (VEGFR2) is a TK receptor expressed by vascular endothelial cells from TME via immune cells, and its activation can initiate HIF1α in tumors and promote VEGF-α overexpression [177, 178]. VEGF-α is mainly expressed in ATC cells but not in normal thyroid tissues and upregulates the PI3K/Akt and MAPK pathways through growth factor signals [179]. In DTC, VEGF and VEGFR2 are overexpressed and can promote tumor progression and invasion. VEGF receptor is also overexpressed in MTC [180]. PI3K/Akt/mTOR and Raf/MEK/ERK are involved in ATC dedifferentiation and tumor growth [181]. TKIs targeting RET and VEGFR2 have shown promising results in phase II trials [182, 183]. Therefore, the study of TKIs in the treatment of TC plays a positive role in improving the current situation of TC patients.

ATA guidelines recommend that patients with stable or minimal progression should not be treated immediately and TKI treatment should be considered in “patients with metastatic, rapidly progressive, symptomatic, and/or imminently threatening disease [184, 185]. Several tyrosine kinase inhibitors have entered clinical trials: (1) sorafenib (BAY 43-9006) can inhibit RAF, VEGFR2, VEGFR3, and KIT kinase, which inhibits TC growth through anti-proliferation and anti-angiogenesis mechanisms [186, 187]; (2) Sunitinib (SU011248) preferentially inhibits VEGFR1-3, KIT, and PDGFR kinase [188]. Sunitinib inhibits the autophosphorylation of RET/PTC and the activation of STAT3, and blocks the transformation ability of RET/PTC [189]; (3) Vandetanib (ZD6474) is an effective inhibitor of VEGFR2, VEGFR3, RET, and epidermal growth factor receptor kinase [190]; (4) Lenvatinib (E7080) inhibits FGFR1-4, PDGFRβ, Vegfr1-3, RET, and supporting element kinase [191]; and (5) Cabotanni (XL184) inhibits c-Met, VEGFR1, 2, and RET kinases [192]. ZD6474 and XL184 have been approved as targeted treatments for advanced MTC with symptoms or high tumor burden [8, 193]. Research has shown that long-term medication cessation in patients may not lead to rapid disease progression. However, it may result in long-term “TKI free” stable diseases in individual patients [194]. Analysis of calcitonin and CDT is necessary during discontinuation to reveal tumor progression. In the event of progress, the same TKI can be used to restart [195]. A large number of studies have demonstrated that TKIs represent a new targeted therapy for invasive, progressive, and refractory TCs [196]. However, there are toxic reactions to inhibiting VEGF treatment, such as hypertension, kidney injury, bleeding, cardiovascular toxicity, etc. [197]. Doctors should closely understand the toxicity, adopt appropriate treatment strategies, and decide on treatment interruption, dosage adjustment, and cessation as needed.

#### LDHA inhibitors

LDHA, one of the pivotal glycolytic enzymes, promotes the conversion of pyruvate to lactic acid by compensating for the reduction in oxidative mitochondrial function and sustains cell survival under hypoxia [117, 198]. The decrease in glucose uptake caused by LDHA inhibitors is not the cause of decreased cell density or reduced GLUT1 surface expression [199]. LDHA inhibitors inhibit the regeneration of nicotinamide adenine dinucleotide (NAD) and impair the activity of glyceraldehyde 3-phosphate dehydrogenase (GAPDH), which accumulates an intermediate volume of glucose in the initial step of glycolysis, increases the cellular level of unused glucose, and inhibits glucose uptake [200]. The overexpression of LDHA and increased phosphorylation are common findings in thyroid malignancies [201]. Patients with high LDHA expression have a poor prognosis, which is closely connected with metastasis, and high LDHA levels have been demonstrated to be related to lymph node metastasis [202]. STAT3 is a new upstream regulator of LDHA and a key transcription factor involved in many growth factors and cytokines, which can trigger various biological processes, including cell growth, differentiation, and survival [116]. The expression of STAT3 is positively associated with the expression of LDHA [203]. The expression levels of STAT3 and PSTAT3 are higher in the group with lymph node metastasis than in the group without lymph node metastasis [204]. LDHA can increase the proliferation, invasion, and metastasis of TC cells and help TC cells evade immunity [100]. Therefore, LDHA is considered as a promising target for the prevention and treatment of TC. Chemical inhibitors of LDHA are being developed, such as the LDHA inhibitors FX11, GSK 2837808A, sodium oxamate, and pyruvic acid, which significantly inhibit cell proliferation and induce apoptosis [205]. Phosphorylated AMPK levels increases when LDHA is knocked down or inhibited. As the primary downstream target of the AMPK signal, mTOR is involved in cell growth, cell proliferation, and cell survival [206]. LDHA knockdown or inhibition reduces the phosphorylation level of mTOR, which also suggests that mTOR inhibitors can be used in the glycolysis process to inhibit the growth and metastasis of TC [202].

#### mTOR inhibitors

mTOR is involved in controlling the proliferation of normal and TC cells and regulating iodide absorption in normal thyroid cells; therefore, mTOR inhibition may efficiently reduce cell proliferation and stimulate iodide absorption in TC cells [207]. mTOR inhibition leads to severe impairment of proliferative signals via the PI3K/Akt pathway [208] and cell cycle arrest in the G1 stage, which can be activated by membrane receptors, including the insulin-like growth factor receptor (IGFR) and the thyroid-stimulating hormone (TSH) receptor in thyroid cells. Rapamycin analogs can directly inhibit mTOR signaling, such as LY294002, AY-22989, AZD8055, and temsirolimus (CCI-7790) [209], and reduce cell proliferation in TC cell lines [210]. Genes encoding TK receptors, mitochondrial activated protein kinase (MAPK) and PI3K/Akt pathway, are mutated in almost all ATC cases [102]. Two open-label phase II clinical trials have demonstrated the modest anti-tumor activity of everolimus and the stabilization of TC [211, 212]. However, neither trial demonstrated an association between tumor mutation status and drug response in patients with ATC. Therefore, targeting these two pathways at the same time may be particularly effective in the treatment of ATC. A PI3K inhibitor (LY294002) was found to inhibit mTOR, slow disease progression, eliminate lung metastasis and prolong the survival time in mice due to inhibition of cancer growth and proliferation, increased apoptosis, and decreased cell activity [213]. The growth inhibition of cancer cell lines treated with MAPK kinase (MEK) and mTOR inhibitor was greater than 60% [111]. Another study demonstrated the therapeutic potential of the novel MEK inhibitor RDEA119 in TC and its synergistic effect with the mTOR inhibitor temsirolimus [214].

#### MEK inhibitors

The MAPK-MEK signaling pathway is often overactivated in ATCs and correlated with the progression of ATCs [215]. MEK inhibitors can induce iodine uptake and retention in TCs, which exhibits G0/G1 arrest by downregulating MEK/ERK phosphorylation and inhibiting the viability of BRAF mutant cells [216, 217]. The MAPK pathway is an evolutionarily preserved signaling cascade that links extracellular and internal stimuli with the control of multiple cellular processes under physiological and pathological conditions, including cell proliferation, survival, invasion, migration, and differentiation [216]. Trametinib, an MEK1/2 inhibitor, has been demonstrated to independently improve survival in patients with metastatic melanoma [218]. Downstream MEK inhibition can not only prevent BRAF resistance in BRAF mutant cells but also block abnormal MAPK activation in BRAF wild-type cells [219]. Pretreatment with MAPK inhibitors improves the reactivity of RAI treatment [220]. MEK inhibitors (such as selumetinib) activate PI3K and MAPK pathways by stimulating HER3 gene expression, and the HER3 inhibitor lapatinib can prevent MAPK rebound and sensitize BRAF^v600E^ positive TC cells to Raf or MAP/ERK inhibitors [221]. The most common adverse effects of selumetinib that were reported are fatigue, diarrhea, and rash [222]. A single-arm multicenter two-phase II clinical trial is currently underway in the UK to evaluate the efficacy of selumetinib in combination with RAI in patients with recurrent thyroid cancer [223]. HER inhibitors combined with BRAF/MEK inhibitors can improve the sensitivity of BRAFv600E positive PTC to BRAF/MEK inhibitors by preventing MAPK rebound and increasing NIS expression [224, 225]. MEK has many unique biochemical and biological characteristics, rendering it an attractive target from the perspective of anticancer drug development.

#### Other strategies

In addition to the above treatments targeting specific enzymes, some studies have found that aerobic exercise may also be a new treatment. Aerobic exercise, an anti-Warburg maneuver, such as swimming and jogging, can increase mitochondrial function and lactate clearance, which increases fat oxidation, decreases glycolysis and reduces dependence on glycogen and glucose [226]. In addition, exercise can reduce the harmful activity of c-Myc [227]. Hence, aerobic exercise helps counteract the metabolic conversion of cancer cells to glycolytic metabolism and produces epigenetic responses that help restore the oxidative phenotype [228]. Other studies have also proved that diets might affect the tumor growth and be a potential treatment [229]. Tailed diets are based on the nutritional vulnerabilities of tumor. Although lacking well-designed clinical trials, some preclinical studies have demonstrated that tailed diet such as low-carbohydrate diet and restring dietary serine and glycine can starve tumors and boots the effectiveness of cancer therapy [230]. Thus, alteration of cellular metabolism by low-carbohydrate ketogenic diets can be an important therapeutic strategy to selectively kill cancer cells that mainly survive on glycolysis [231]. Calorie-restricted diets enhance ameliorate metabolic pathogenesis and reduce the incidence of cancer [232, 233]. Also, caloric restriction promotes antineoplastic immune responses and suppresses tumor cell proliferation [234, 235]. Hence, metabolic interventions may have a great potential as co-adjuvant therapy in the management of TC.

## Conclusions

The increasing incidence rate of TC has been a significant concern in the medical field. The unambiguous pathogenesis of TC is not yet fully understood because of its diversity. Glycolysis is a process that occurs in all cancer cells. Linking it with TC provides some insights for the treatment of TC. Warburg effect, that is, aerobic glycolysis in the presence of oxygen and mitochondria with normal function in principle, constitutes the main driving factor of cancer progression mechanism, resistance to traditional therapy and poor prognosis of patients. The molecular and functional processes associated with tumorigenesis may include: (a) significant acceleration of glycolytic flux; (b) generation of sufficient ATP to provide energy for cancer cells; (c) backup and transfer of glycolytic intermediates, promoting the biosynthesis of nucleotides, nonessential amino acids, lipids and hexosamine; (d) inhibition of pyruvate from entering mitochondria; (e) excessive formation and accumulation of lactate; (f) maintaining cell redox homeostasis and low ROS formation; and (h) HIF-1 overexpression, mutant p53 and mutant PTEN, which inhibit mitochondrial biogenesis and function. The Warburg effect can help cancer cells survive, grow and metastasize, further helping tumors resist apoptosis, and avoid destruction by the immune system. Glycolysis has a complete mechanism. By understanding the process of its occurrence and comparing differences in glycolysis processes between normal cells and cancer cells, we can target the glycolysis pathway to treat TC in follow-up research. The common treatment for TC is surgical resection, but recurrence or deterioration is still possible. For special types of TC, the current treatment cannot achieve a good therapeutic effect, and whether we can target glycolysis to achieve a therapeutic effect requires further exploration.

With improved understanding of “reprogramming of glucose metabolism” in TC, patients with poorly differentiated TC are no longer without effective therapies in terms of the development of new therapies. Novel diagnostic methods based on glycolysis mechanism, such as FDG-PET, as well as targeting drugs, such as FX11, trametinib, and AZD8055, and diets will be of great significance to further deepen our understanding of glycolysis regulation and reasonably design strategies for the diagnosis and treatment of TC, especially for patients with poorly differentiated TC or relapse status. Many inhibitors have entered the stage of clinical experimental research, but no extraordinary evidence shows that they have a good therapeutic effect. Research in this field still needs to be further strengthened. Moreover, how to affect the occurrence and development of TC requires further verification. Whether inhibitors affecting the glycolysis pathway have a definitive inhibitory effect on TC and their safety warrants our attention. Taken together, targeting the cancer metabolism holds great promise as a therapeutic modality in TC.

## Data Availability

This article contains data to support the results of this study. The datasets generated and/or analyzed during the current study are not publicly available due to participant information privacy but are available from the corresponding author on reasonable request.

## Abbreviations

TC: Thyroid cancer
DTC: Differentiated thyroid cancers
PTC: Papillary thyroid cancer
FTC: Follicular thyroid cancer
MTC: Medullary thyroid cancer
ATC: Anaplastic thyroid cancer
RAIR: Radioiodine refractory
ATP: Adenosine triphosphate
PI3K: Phosphatidylinositol 3-kinase
HIF-1α: Hypoxia-inducible factor-1 alpha
TME: Tumor microenvironment
PK: Pyruvate kinase
GLUT1: Glucose transporter 1
TCA cycle: Tricarboxylic acid cycle
LA: Lactic acid
PEP: Phosphoenolpyruvate
GLS: Glutaminase
GDH: Glucose dehydrogenase
PFK: Phosphofructokinase
ROS: Reactive oxygen species
LDHA: Lactate-dehydrogenase A
PD-1: Programmed cell death protein 1
PGC-1: Peroxisome proliferator-activated receptor γ coactivator-1
MAPK: Mitogen-activated protein kinase
MCTs: Proton-linked monocarboxylate transporters
ACF: Acriflavine
CO2: Carbon dioxide
PPP: Pentose phosphate pathway
ECM: Extracellular matrix
BM: Basement membrane
EMT: Epithelial-mesenchymal transition
MMP: Matrix metalloproteinases
PD-L1: Programmed death-ligand 1
FNAB: Fine-needle aspiration biopsy
CT: Computed tomography
MRI: Magnetic resonance imaging
FDG-PET: Fluoro-2-deoxy-D-glucose positron emission tomography
NIS: Na + /I- symporter
ATA: American Translators Association
Tg: Thyroglobulin
DWI: Diffusion-weighted imaging
TKIs: Tyrosine kinase inhibitors
VEGFR2: Vascular endothelial growth factor receptor 2
VEGF: Vascular endothelial growth factor
PDGFR: Platelet-derived growth factor receptors
STAT3: Signal transducer and activator of transcription 3
NAD: Nicotinamide adenine dinucleotide
GAPDH: Glyceraldehyde 3-phosphate dehydrogenase
IGFR: Insulin-like growth factor receptor
TSH: Thyroid stimulating hormone
